# Supplementary material for: CDK5RAP3 Deficiency Is Associated with Hepatic Inflammation and Increased Expression of NLRP3 Inflammasome Components
Source: Biomedicines. 2025 Aug 21;13(8):2030. doi: 10.3390/biomedicines13082030 (PMC12383291; doi:10.3390/biomedicines13082030)
Supplement: Supplementary file 1 [file biomedicines-13-02030-s001.zip › File S1 Supplementary Materials and Methods.pdf]

Supplementary Materials and Methods

Crystal violet staining

In 60 mm dishes, the number of cells in the first inoculation was the same. Collect samples every 24 h (24 h, 48 h, 72 h, 96 h), and after washing 3 times in PBS, it was fixed in 4% paraformaldehyde for 20 min. Remove paraformaldehyde, cells were washed 3 times with ddH_2O. Then, stain it for 20 min with 0.1% crystal violet (Macklin, 548-62-9, Shanghai, China). After aspirating the crystal violet and washing it three times with water, let it air dry, then add 2 mL 10% acetic acid to each well and shake for 20 min.

Apoptosis detection using the Flow Cytometry

Annexin V-FITC/PI Apoptosis Detection Kit (Vazyme, A211, China) was performed, according to the manufacturer's instruction. Digest MEFs with trypsin (Solarbio, T1350, China) without EDTA and incubate the cells with FITC and PI in Binding Buffer for 10 min at room temperature in the dark. Detect with flow cytometer (Becton Dickinson, Mountain View, CA) within 1 h. Data were analyzed using Flow 10 software. Live cells were recorded as a combination of Annexin V-FITC$^-$/PI$^-$, the remaining cells were counted as apoptotic cells (early apoptosis cells were recorded as a combination of Annexin V-FITC$^+$/PI$^-$, and late apoptosis cells were recorded as a combination of Annexin V-FITC $^+$/PI$^+$, and necrosis cells were recorded as a combination of Annexin V-FITC $^-$/PI$^+$).
