# Supplementary material for: CDK5RAP3 Deficiency Is Associated with Hepatic Inflammation and Increased Expression of NLRP3 Inflammasome Components
Source: Biomedicines. 2025 Aug 21;13(8):2030. doi: 10.3390/biomedicines13082030 (PMC12383291; doi:10.3390/biomedicines13082030)
Supplement: Supplementary file 1 [file biomedicines-13-02030-s001.zip › Table S1 Antibodies used in this paper.pdf]

Table S1 Antibodies used in this paper

Primary antibodies	Vendor	Dilution	Source
IL6(IF/WB)	Abcam (ab9324)	1:200/1:2000	Mouse
IL1β(WB)	ABclonal (A23416)	1:2000	Rabbit
GSDMD(WB)	ABclonal (A17308)	1:1000	Rabbit
Caspase1(WB)	Proteintech (22915-1-AP)	1:2000	Rabbit
BAX(WB)	Proteintech (50599-2-Ig)	1:2000	Rabbit
TNF-alpha(WB)	Proteintech (17590-1-AP)	1:1000	Rabbit
CDK5RAP3(WB)	Abcam (ab157203)	1:2000	Rabbit
Bcl-2(WB)	ABclonal (A0208)	1:1000	Rabbit
NLRP3(IF/WB)	Proteintech (19771-1-AP)	1:200/1:1000	Rabbit
Alb (IF)	Proteintech（16475-1-AP）	1:200	Mouse
CDK5RAP3(IF)	Abcam(ab168353）	1:200	Rabbit
**Secondary antibodies**			
**Goat Anti-Rabbit IgG H&L (Alexa Fluor® 488)(IF)**	**Abcam (ab150077)**	**1:150**	**Goat**
**Goat Anti-Mouse IgG H&L (Alexa Fluor® 647)(IF)**	**Abcam(ab150115)**	**1:150**	**Goat**
**HRP-conjugated Goat Anti-Rabbit IgG(H+L)(WB)**	**Proteintech(AB_2722564)**	**1:5000**	**Goat**
**HRP-conjugated Goat Anti-Mouse IgG(H+L)(WB)**	**Proteintech(AB_2722565)**	**1:5000**	**Goat**
