# Supplementary material for: CDK5RAP3 Deficiency Is Associated with Hepatic Inflammation and Increased Expression of NLRP3 Inflammasome Components
Source: Biomedicines. 2025 Aug 21;13(8):2030. doi: 10.3390/biomedicines13082030 (PMC12383291; doi:10.3390/biomedicines13082030)
Supplement: Supplementary file 1 [file biomedicines-13-02030-s001.zip › Table S2 Primers used for quantitative RT.pdf]

Table S2 Primers used for quantitative RT-PCR

Genes	Genbanks	Forward primer sequences	Reverse primer sequences	Product Length (bp)
NLRP3	NM_001359676.1	GACCAGCCAGAGTGGAATGA	CTTCAAGGCTGTCCTCCTGG	92
TNFα	NM_013693.3	CCCTCACACTCAGATCATCTTCT	GCTACGACGTGGGCTACAG	61
IL1β	NM_008361.4	GAAATGCCACCTTTTGACAGTG	TGGATGCTCTCATCAGGACAG	118
IL6	NM_031168.2	AGACAAAGCCAGAGTCCTTCAG	GTGACTCCAGCGTATCTCTTGGT	148
Bax	NM_007527.3	ATGCGTCCACCAAGAAGCTGAG	CCCCAGTTGAAGTTGCCATCAG	102
Bcl2	NM_009741.5	GCAGAGATGTCCAGTCAG	CACCGAACTCAAAGAAGG	94
CDK5RAP3	NM_001308183.1	ATGAGATCGACTGGGGTGAC	AGCCTCAGTTCCTGTCTCCA	156
β-Actin	NM_007393.5	TGCTGTCCCTGTATGCCTCTG	GGTGTAAAACGCAGCTCAGTAA	245
ASC	NM_023258.3	CTGCTCAGAGTACAGCCAGAAC	CTGTCCTTCAGTCAGCACACTG	181
